# Rapid evolutionary divergence of *Gossypium barbadense* and *G. hirsutum* mitochondrial genomes

**DOI:** 10.1186/s12864-015-1988-0

**Published:** 2015-10-12

**Authors:** Mingyong Tang, Zhiwen Chen, Corrinne E. Grover, Yumei Wang, Shuangshuang Li, Guozheng Liu, Zhiying Ma, Jonathan F. Wendel, Jinping Hua

**Affiliations:** Department of Plant Genetics and Breeding /Key Laboratory of Crop Heterosis and Utilization of Ministry of Education /Beijing Key Laboratory of Crop Genetic Improvement, China Agricultural University, Beijing, 100193 China; Department of Ecology, Evolution and Organismal Biology, Iowa State University, Ames, IA50011 USA; Institute of Cash Crops, Hubei Academy of Agricultural Sciences, Wuhan, 430064 Hubei China; College of Agronomy, Hebei Agricultural University, Baoding, 071001 Hebei China; Present address: Saskatchewan Cancer Agency, Division of Oncology, Department of Biochemistry, University of Saskatchewan, Saskatoon, Saskatchewan Canada; Present address: Department of Breeding Research, Leibniz Institute of Plant Genetics and Crop Plant Research (IPK), OT Gatersleben, Corrensstrasse 3, D-06466 Stadt, Seeland Germany

**Keywords:** Mitochondrial genome, Comparative genomics, DNA rearrangement, Duplicated copy, Sequence transfer

## Abstract

**Background:**

The mitochondrial genome from upland cotton, *G. hirsutum*, was previously sequenced. To elucidate the evolution of mitochondrial genomic diversity within a single genus, we sequenced the mitochondrial genome from Sea Island cotton (*Gossypium barbadense* L.).

**Methods:**

Mitochondrial DNA from week-old etiolated seedlings was extracted from isolated organelles using discontinuous sucrose density gradient method. Mitochondrial genome was sequenced with Solexa using paired-end, 90 bp read. The clean reads were assembled into contigs using ABySS and finished via additional fosmid and BAC sequencing. Finally, the genome was annotated and analyzed using different softwares.

**Results:**

The *G. barbadense* (Sea Island cotton) mitochondrial genome was fully sequenced (677,434-bp) and compared to the mitogenome of upland cotton. The *G. barbadense* mitochondrial DNA contains seven more genes than that of upland cotton, with a total of 40 protein coding genes (excluding possible pseudogenes), 6 rRNA genes, and 29 tRNA genes. Of these 75 genes, *atp1*, *mttB*, *nad4*, *nad9*, *rrn5*, *rrn18*, and *trnD(GTC)-cp* were each represented by two identical copies. A single 64 kb repeat was largely responsible for the 9 % difference in genome size between the two mtDNAs. Comparison of genome structures between the two mitochondrial genomes revealed 8 rearranged syntenic regions and several large repeats. The largest repeat was missing from the master chromosome in *G. hirsutum*. Both mitochondrial genomes contain a duplicated copy of *rps3* (*rps3*-2) in conjunction with a duplication of repeated sequences. Phylogenetic and divergence considerations suggest that a 544-bp fragment of *rps3* was transferred to the nuclear genome shortly after divergence of the A- and D- genome diploid cottons.

**Conclusion:**

These results highlight the insights to the evolution of structural variation between Sea Island and upland cotton mitochondrial genomes.

**Electronic supplementary material:**

The online version of this article (doi:10.1186/s12864-015-1988-0) contains supplementary material, which is available to authorized users.

## Background

Plant mitochondrial genomes are remarkable from both evolutionary and comparative genomics stand-points. Like their animal counterparts, plant mitochondrial genomes generally are characterized as circular chromosomes [1] (barring notable exceptions, e.g., [2, 3]) that contain a variable number of genes interspersed within non-coding DNA; however, this simplistic generalization belies the dynamic and complex nature of plant mitochondrial genomes [4]. Not only is the overall structure mitochondrial genomes an oversimplification of their possible morphologies [5–7], but recent comparative analyses among flowering plants have demonstrated extensive fluidity in plant mitochondrial genomes [2, 8, 9]. The structure and evolution of angiosperm mitochondrial genomes are driven by extremely high rates of recombination and rearrangement, with major rearrangements detected even in hybrid plants [10]. Paradoxically, mitochondrial genes are among the slowest evolving, and this rate paradox can be partially explained by DNA repair mechanisms [11]. DNA repair in the coding regions of the mitochondria is biased toward gene conversion, reducing the mutation rates within genes, whereas the more inaccurate break-induced replication (BIR) is common in the noncoding regions, leading to the expansions and rearrangements observed outside of genes [12–15]. Consequently, plant mitochondrial genomes vary remarkably both in size and composition within plant families and genera [7, 9, 16, 17], with genome sizes ranging from 30 kilobases in some algae to several megabases in certain angiosperms [2, 3, 18]. Intraspecies comparisons suggest that plant mitochondrial genomes can be highly divergent even among different varieties of the same species [19, 20], and together with the observed genomic diversity within a single order of angiosperms [21], further indicates the remarkable diversity in mitochondrial genomes among green plants [22].

Perhaps the two most surprising recent realizations regarding plant mitochondrial genome evolution are the extensive variability in mitochondrial genome size and the compositional changes that have led to this variability. Plant mitochondrial genomes vary by an amazing 870-fold, from the ultra-compact, 12 kb (12,998 bp) genome (Accession Number: NC 010357) of the alga *Polytomella capuana* [23] to the spectacularly bloated 11,319 kb genome (11,318,806 bp) of *Silene conica* [2]. The evolutionary dynamics that underlie this remarkable variation are not fully understood; however, it is clear from several analyses that plant mitochondrial genomes are repositories for DNA from myriad sources [24]. These not only include the nucleus and chloroplast genomes of the host species itself, but may also include sequences derived from the chloroplast and mitochondrial genomes of other species [3]. Much of this sequence is large (>1 kb) and repetitive in nature [25], providing sufficient tracts of homology to promote the highly dynamic recombination evident in plant mitochondrial genomes [25–27]. Indeed, it is the high rates of sequence acquisition/loss and recombination that give plant mitochondrial genomes their reputation for rapid intergenic evolution, leading to low levels of non-genic homology among even closely related species [2, 8, 28]. Furthermore, this propensity for recombination can have additional intriguing consequences, such as the generation of substoichiometic recombinant molecules [29, 30], variable chromosomal structures [7, 31, 32], and novel cytoplasmic male sterility (CMS)-inducing open reading frames (ORFs) [19, 20, 33, 34].

Despite the extensive variation in sizes and structures of plant mitochondrial genomes, their coding sequences rank among the most slowly evolving genes known [35, 36]. Although considerable gene- and lineage-specific variation in rates of gene retention/loss exist for both protein and tRNA genes [37], most sequenced angiosperm mitochondrial genomes have ~50–60 genes, including subunits of respiratory complexes, ribosomal RNAs (rRNAs), and transfer RNAs (tRNAs) [37], and a variable number of pseudogenized forms and/or copies of mitochondrial genes [38–42].

Sea Island cotton (*Gossypium barbadense* L.) is a New World allotetraploid (2n = 52) grown in many countries because of its superior quality fiber [43]. Upland cotton (*G. hirsutum*), however, is more commonly grown because it is earlier maturing and has a higher yield potential, and accordingly it now accounts for about 90 % of world fiber production. Sea Island cotton (*G. barbadense*) accounts for only approximately 5 % of present global commerce [44]. In addition to its superior spinning performance and unique high quality fiber characteristics, Sea Island cotton is a potential source of genes for resistance to *Verticillium* wilt [45, 46]. The objective of the present study was to complement earlier efforts [47, 48] to generate a high-quality sequence of the mitochondrial genome of *G. barbadense*. We provide this sequence and compare it to the mitogenome of *G. hirsutum* [41], resulting in insights to the evolution of structural variation and new fields into mtDNA duplicated copy gene.

## Methods

### Plant materials and mitochondrial DNA extraction

Mitochondria were isolated from week-old etiolated seedlings of “Pima 90–53”, a variety of Sea Island cotton (*G. barbadense* L.) whose seeds were obtained from Hebei Agricultural University [40, 49]. Mitochondrial DNA was extracted from isolated organelles as reported [40, 41]. Briefly, the extraction protocol for the mtDNA of Sea Island cotton was as follows:

1. The seeds were planted in sand and the seedlings were kept in darkness to obtain etiolated seedlings. From these, 7 d-old etiolated seedlings were ground and used to isolate mitochondria.
2. Ground seedlings were collected and further purified by centrifugation in a discontinuous sucrose-density gradient (60 %, 52 %, 36 % and 20 % M/V) in purification buffer (10 mM Tris–HCl pH 7.4 and 20 mM EDTA) (Additional file 1: Figure S1)
3. The mitochondria band from the interface between 52 % and 36 % was carefully collected and washed with 0.3 mol · L^−1^ sucrose buffer to obtain the intact mitochondrial fractions.
4. The mitochondrail fraction was lysed in cetyltrimethyl ammonium bromide (CTAB) for release of mtDNA, and further purified by proteinase K digestion, phenol-chloroform extraction, and ethanol precipitation.

The plastid band was located in the interface between 36 % and 20 % sucrose, while the nuclei were precipitated to the bottom. PCR validation failed to detect nuclear contamination, but did detect partial contamination from plastid DNA (Additional file 2: Figure S2). To avoid contamination from chloroplast, we filtered the reads based on the sequence of *Gossypium barbadense* chloroplast genome before assembly.

### Mitochondrial genome sequencing and assembly

Isolated Sea Island cotton mitochondrial DNA was cloned into whole-genome shotgun libraries using CopyControl Fosmid Library Production Kit (Epicentre, Cat. No. CCFOS110) and sequenced to about 700 × coverage with Solexa using paired-end, 90 bp read at Beijing Genomics Institute (BGI). Adaptor and contaminant sequences were removed from the raw reads and the clean reads were assembled using ABySS [50]. Since nuclear and chloroplast contamination is possible in the extraction procedure, BLASTn [51] against nt/nr database was used to identify and remove contaminant contigs. In addition, known mitochondrial genome sequences of *G. hirsutum* [41] and *G. harknessii* (unpublished) were also used to identify mitochondrial-type contigs. Contigs were ordered/oriented and gaps were closed via additional fosmid and BAC sequencing. Primers representing both conserved mitochondrial genes and scaffold terminals were used to screen both a fosmid library [40] and a BAC library [48, 49]. Twenty fosmid clones (also previously associated with *G. barbadense* mitochondria; see Fig. 5 in [40]) and two BAC clones were selected by this PCR screen and independently sequenced by Solexa and 454 sequencing methods in BGI and Shanghai Majorbio Bio-pharm Biotechnology, respectively. The resulting clones were assembled with SOAPdenovo [52] and Newbler (Version 2.53), respectively; these were then used to anchor and orient the previously assembled mitochondrial contigs into supercontigs. To close the remaining gaps, the known relationships of the fosmids were used to predict the order and orientation of contigs, and the remaining gaps were filled by LA-PCR (Long and Accurate Polymerase Chain Reaction) using the primers listed in Additional file 3: Table S1. These primers were also used to verify each contig joined.

### Genome annotation and sequence analysis

**Fig. 1:**
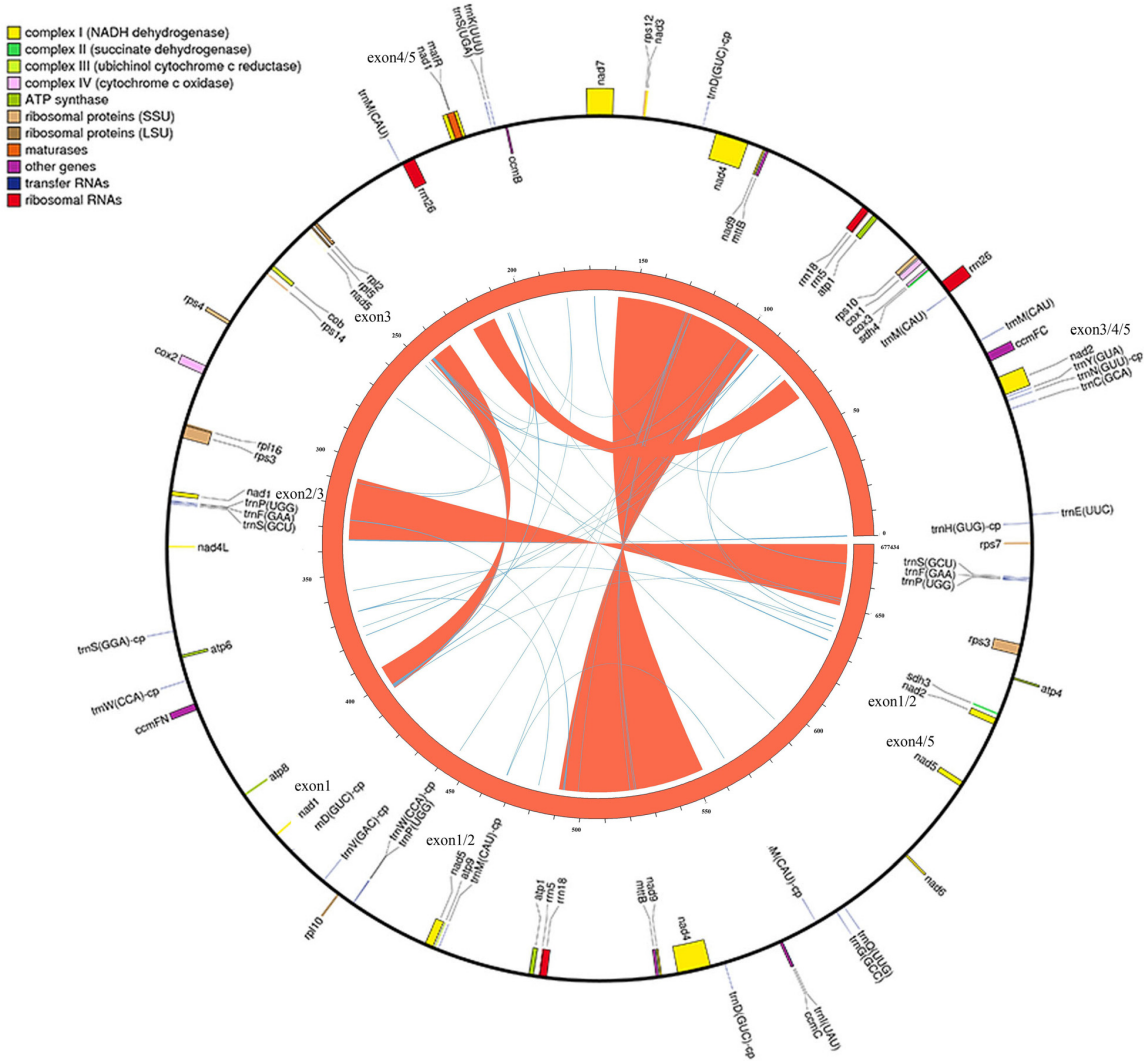
Genome map of *Gossypium barbadense* mitochondrial genome. The map shows both the gene map (outer circle) and repeat map (inner map). Genes exhibited on the inside of outer circle are transcribed in a clockwise direction, while genes on the outside of outer circle are transcribed in a reverse direction. The inner circle reveals the distribution of repeats in *G. barbadense* mt genome with curved lines and ribbons connecting pairs of repeats and width proportional to repeat size. The red ribbons represent > = 1 Kb repeats and the blue lines represent repeats between 100 bp to 1 Kb. The numbers give genome coordinates in kilobases

Mitochondrial genes were annotated as reported [16], using the genes annotated in the *G. hirsutum* mtDNA as references. Functional genes (other than tRNA genes) were identified by local blast searches against the database, whereas tRNA genes were predicted *de novo* using tRNAscan-SE [53]. A genome map (Fig. 1) was generated using OGDRAW [54] and the repeat map was drawn by Circos [55].

The newly generated *G. barbadense* sequence was aligned to the published *G. hirsutum* mitochondrial sequence [41], and the values of dS and dN/dS were evaluated with PAML4 [56]. PipMaker was used to identify repeated sequences within *G. barbadense* [57], and repetitive DNA from nuclear sources was identified using RepeatMasker (http://www.repeatmasker.org) and a custom, *Gossypium*-enriched repeat database. Dot matrix comparisons were generated between the mitochondrial genome of *G. barbadense* and those of *Arabidopsis thaliana*, *Carica papaya*, and *G. hirsutum* using the nucmer program of MUMmer with the parameters: 100-bp minimal size for exact match and 500-bp minimal interval between every two matches [58]. We used Circos plots [55] to show the collinear relationships between *G. barbadense* and *G. hirsutum* mitochondrial genome sequences. Possible pseudogenes and non-functional tRNAs were predicted using previously published mitochondrial genomes, and the distribution of pseudogenes was drawn by program pheatmap in R. A phylogenetic tree was constructed based on 17 conserved mitochondrial genes (*nad1*, *nad2*, *nad3*, *nad4*, *nad4L*, *nad5*, *nad6*, *nad9*, *cob*, *cox1*, *cox2*, *cox3*, *atp1*, *atp4*, *atp6*, *atp8*, *atp9*) using maximum likelihood (ML) method with the model GTR + G + I in MEGA5.05 [59].

## Results and discussion

### Assembly of the complete *G. barbadense* mitochondrial genome

A total of 607 Mbp (sequence coverage: 867×) of clean reads was generated for the *G. barbadense* mitochondrial genome. These reads were initially assembled into 14 contigs (average length = 43,530 bp; putative contaminant contigs removed), ranging in size from 10,246 bp to 105,651 bp. Because repeated sequences hinder the assembly of these contigs into a single circular chromosome, 20 Fosmid clones and two BAC clones were sequenced and used to inform the order and orientation of these contigs. In addition, the previously published physical map of the *G. barbadense* mitochondrial genome was also used [40]. The order and orientation of contigs was confirmed and remaining gaps were filled using PCR (Additional file 3: Table S1). The Sea Island mitochondrial genome was assembled as a 677,434 bp circular molecule with four large repeats (Fig. 1) (Genbank Accession Number KP898249), similar to an earlier prediction of mitogenome size (690–700 kb) [60].

### Comparative analysis of *G. barbadense* and *G. hirsutum* mitochondrial genomes

The mitochondrial genomes of *G. barbadense* and *G. hirsutum* [41] are largely similar; however, as observed in other genera, many differences exist even between these closely related species (Table 1). The size difference between the mitochondrial genomes of *G. barbadense* and *G. hirsutum* is about 9 %, representing almost 56 kb of additional sequence in *G. barbadense.* In terms of nucleotide composition, the two mitochondrial genomes are almost identical, with the GC content of *G. barbadense* and *G. hirsutum* being 44.98 % and 44.95 %, respectively. Likewise, a similar number of genes were predicted for both, with *G. barbadense* having seven more functional genes annotated than did *G. hirsutum* (75 versus 68 genes, respectively; Table 2), including 4 additional protein coding genes, 2 additional rRNA genes, and one more tRNA gene, generating a slightly higher gene length in the *G. barbadense* than in the *G. hirsutum* mitochondrial genome (36.4 kb versus 31.7 kb). In total, 40 protein coding genes, 6 rRNA genes, and 29 tRNA genes were predicted for *G. barbadense*. Most of these genes were intact, even in the duplicate copies; however, both the sole *nad1* and the *rps3* copy displayed deviations from expectations for intact genes (compared to *G. hirsutum*, the *G. barbadense* mitochondrial genome contains extra *nad1b* and *nad1c* exons. The truncated *rps3* is 544 bp shorter than the intact copy in *G. hirsutum*.).

**Table 1:** General features of mitochondrial genomes of *G. barbadense* and *G. hirsutum*

Genome features	*G. barbadense*	*G. hirsutum*
Total genome size (bp)	677,434	621,884
GC content (%)	44.98	44.95
Total repeated sequences (bp)^a^	236,070	132,305
Percentage of genome (%)	(34.85)	(21.27)
*gypsy*-like	12,118	10,013
*copia*-like	5.778	5,583
unclassified LTR-retrotransposon	11,954	9,662
unclassified retroelement	3,011	2,591
*Mutator*-like	1,076	1,590
unclassified transposable element	6,928	5,791
unclassified	195,205	97,075
Chloroplast-like sequences (bp)	5,383	6,833
Percentage (%)	(0.80)	(1.10)
Coding sequences (bp)^b^	36,365	31,721
Percentage (%)	(5.37)	(5.10)
Gene content^b^	75	68
Protein-coding genes^c^	40	36
rRNA	6	4
tRNA	29	28

^a^Repeats > 100 bp

^b^All copies of duplicated genes but not pseudogenes are included

^c^The extra protein-coding genes in *G. barbadense* are *atp1*, *mttB*, *nad4* and *nad9*

**Table 2:** Genes identified in the *G. barbadense* mitochondrial genome

1. Complex I genes	6. Cytochrome c biogenesis genes	11. rRNA genes	*trnP(TGG)*
*nad1*	*ccmB*	*rrn5-1*	*trnM(CAT)-cp-1*
*nad2*	*ccmC*	*rrn5-2* ^a^	*trnSup(TTA)-psu-2*
*nad3*	*ccmFC*	*rrn18-1*	*trnD(GTC)-cp-2* ^a^
*nad4-1*	*ccmFN*	*rrn18-2* ^a^	*trnI(TAT)*
*nad4-2* ^a^	7. Ribosomal proteins, LSU	*rrn26-1*	*trnM(CAT)-cp-2*
*nad4L*	*rpl2*	*rrn26-2*	*trnG(GCC)*
*nad5*	*rpl5*	12. tRNA-coding genes	*trnQ(TTG)*
*nad6*	*rpl10*	*trnH(GTG)-cp*	*trnP(TGG)-2*
*nad7*	*rpl16*	*trnE(TTC)*	*trnF(GAA)-2*
*nad9-1*	8. Ribosomal proteins, SSU	*trnC(GCA)*	*trnS(GCT)-2*
*nad9-2* ^a^	*rps3*	*trnN(GTT)-cp*	
2. Complex II genes	*rps3-psu*	*trnY(GTA)*	
*sdh3*	*rps4*	*trnM(CAT)*	
*sdh4*	*rps7*	*trnM(CAT)-1*	
3. Complex III gene	*rps10*	*trnSup(TTA)-psu-1*	
*cob*	*rps12*	*trnD(GTC)-cp-1*	
4. Complex IV genes	*rps14*	*trnK(TTT)*	
*cox1*	9. Maturase gene	*trnS(TGA)*	
*cox2*	*matR*	*trnM(CAT)-2*	
*cox3*	10. protein translocation system subunit gene	*trnP(TGG)-1*	
5. Complex V genes	*mttB-1*	*trnF(GAA)-1*	
*atp1-1*	*mttB-2* ^a^	*trnS(GCT)-1*	
*atp1-2* ^a^		*trnS(GGA)-cp*	
*atp4*		*trnW(CCA)-cp-1*	
*atp6*		*trnD(GTC)*	
*atp8*		*trnV(GAC)-cp*	
*atp9*		*trnW(CCA)-cp-2*	

A total of 40 protein-coding genes, 6 rRNA-coding genes and 29 tRNA-coding genes were identified (excluding pseudogenes), in addition to 3 pseudogenes and 10 chloroplast-derived sequences. Genes present in duplicate are denoted with a hyphenated number (e.g., −1 or −2). ^a^ represents the second gene copy to *G. barbadense*

As with the annotated genes, the amount of chloroplast-derived sequence was similar between the two mitochondrial genomes, with *G. barbadense* having 1.42 kb less identifiable chloroplast-derived sequence (Table 1). In *G. barbadense*, 19 fragments ranging from 35 bp to 2,203 bp in size, contribute 5,383 bp of sequence to the genome (>1 %; Table 1 and Additional file 4: Table S2) versus 6,833 bp in *G. hirsutum*. Most of the inserted sequences in both cases were either non-coding or were tRNAs. With respect to tRNAs, both have nearly the same set of tRNAs; however, *G. barbadense* has additional copy of *trnD(GTC)-cp*, but lacks one of the five conserved cp-derived tRNAs [41] (chloroplast-derived *trnP*).

Together, the differences between the two cotton mitochondrial genomes attributable to gene or chloroplast-derived sequence represent a small fraction of the difference in genome size (~5 % of the total size difference). As expected from the nature of plant mitochondrial genomes, the greatest difference was in the proportion of repeated sequences, with approximately 1.8 times more sequence in *G. barbadense* derived from repetitive sequences than in *G. hirsutum* (21.27 %). Interestingly, the amount of sequence attributable to identifiable transposable elements comprised only 17.3 % and 26.6 % of the repetitive sequences detected in the *G. barbadense* and *G. hirsutum* mitochondrial genomes, respectively. The remainder of the sequence was unclassified repetitive sequences contained within the mitochondrial genomes themselves. As with nuclear genomes, *gypsy* elements comprised the largest fraction of the identifiable repetitive sequences, and followed by unclassified LTR-retrotransposons and transposable elements.

The presence and distribution of short repeats also distinguished the two mitochondrial genomes, with 207 and 343 repeats larger than 19 bp in *G. barbadense* and *G. hirsutum*, respectively (Table 3). As in *G. hirsutum*, *G. barbadense* short repeats were typically small (20 bp to 39 bp) [41]. Therefore, while the short repeats were more numerous, their small length had relatively little effect compared to the large repeats (>10 kb; average size in *G. barbadense* = ~28 kb) (Fig. 1 and Additional file 5: Table S3). In fact, most of the genome expansion in *G. barbadense* is attributable to the largest repeat (R1 = 63,904 bp), contributing a full 18.9 % of the genome, as well as several duplicated rRNA genes (*rrn5* and *rrn18*). Such large repeats have precedence in plant mitochondrial genomes, including, for example, a 120-kb repeat in maize [5] and an 87-kb repeat in *Beta* [61]. In total, the proportion of repeats in *G. barbadense* was nearly 1.5 times that of *G. hirsutum* (Table 3).

**Table 3:** Frequency distribution of repeat lengths in the mitogenomes of *G. hirsutum* and *G. barbadense*

Size (bp)	Number		Total length of repeats (bp)		Coverage (%)	
	*G. hirsutum*	*G. barbadense*	*G. hirsutum*	*G. barbadense*	*G. hirsutum*	*G. barbadense*
20–39	192	55	10,747	4,281	1.7	0.6
40–59	69	83	9,667	10,139	1.6	1.5
60–79	35	29	9,567	5,183	1.5	0.8
80–99	11	12	8,365	2,194	1.3	0.3
100–1,000	32	24	18,368	12,670	3.0	1.9
>10 K	4	4	117,300	223,400	18.9	33.0

### Syntenic regions and rearrangement

**Fig. 2:**
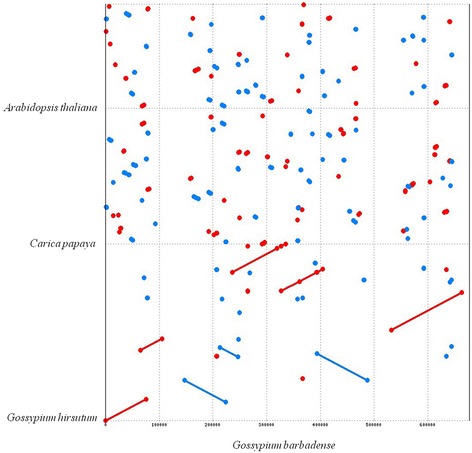
Dot matrix analyses between *G. barbadense* and *G. hirsutum*, *C. papaya*, *A. thaliana* (individually) by whole genomic alignment. The blue and red lines refer inverted and direct syntenic regions, respectively

**Fig. 3:**
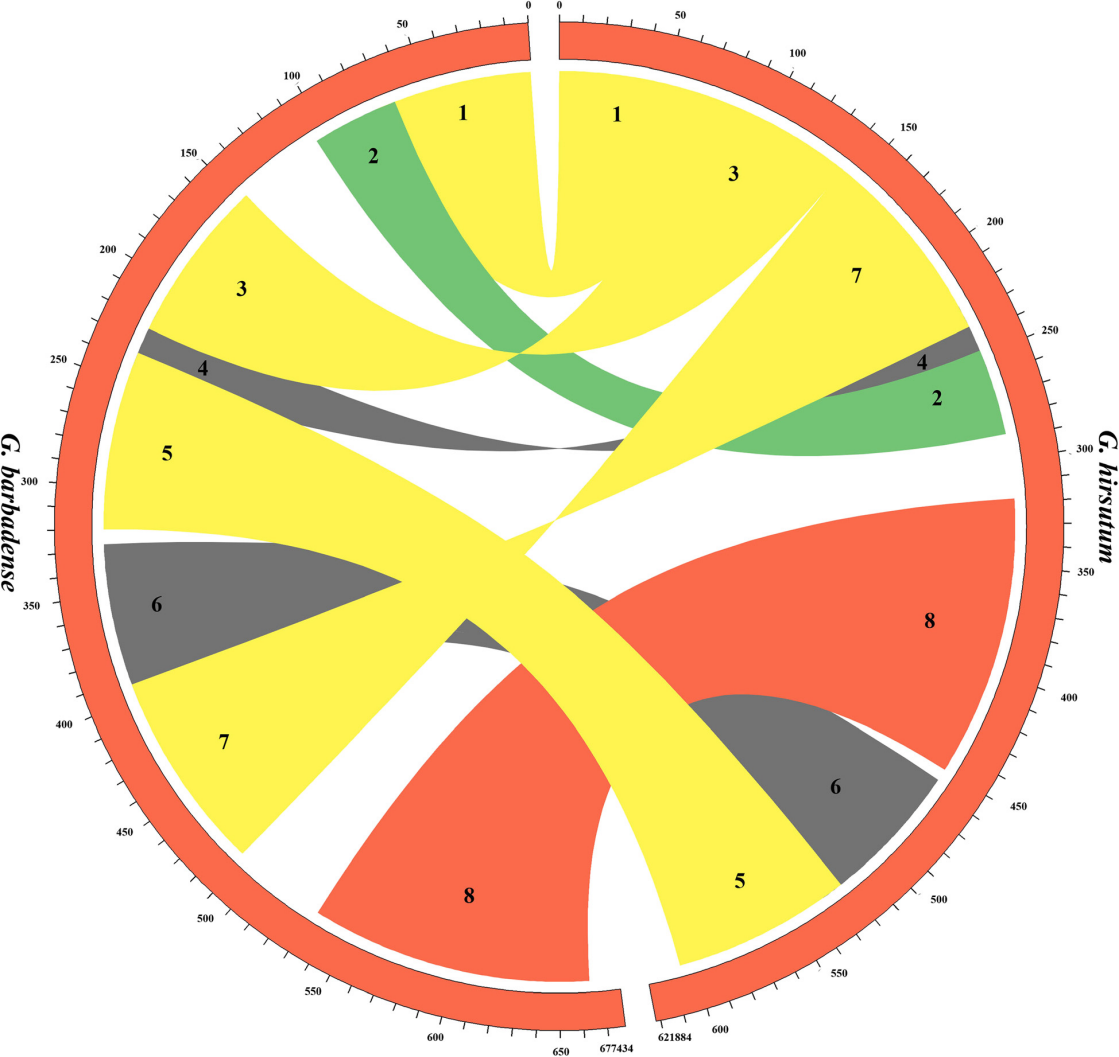
Syntenic blocks larger than 10 Kb between *G. barbadense* and *G. hirsutum* with curved ribbons connecting pairs of syntenic blocks and width proportional to blocks size. The numbers give genome coordinates in kilobases

Syntenic regions were identified between *G. barbadense* and *A. thaliana*, *C. papaya*, and *G. hirsutum*, respectively. Plant mitochondrial genomes are known to experience myriad synteny-disrupting rearrangements over short evolutionarily timescales, and, reflecting this, appreciable synteny was limited to the *G. barbadense* - *G. hirsutum* comparison (Fig. 2). A set of 8 sequence blocks larger than 10 Kb with high homology (>99.8 %) were detected between the *G. barbadense* and *G. hirsutum* mitochondrial genomes, here named block 1 to block 8, respectively (Additional file 6: Table S4). The sizes of these eight syntenic blocks ranged from 33.0 kb (block 4; Fig. 3) to 131.5 kb (block 8; Fig. 3). Interestingly, after the four large repeats (R1-R4) were identified on the *G. barbadense* mitochondrial genome (Fig. 1 and Additional file 7: Figure S3), we also found a short direct repeat “R08” (Additional file 5: Table S3) at the ends of large repeat R1 (Fig. 1 and Additional file 7: Figure S3). Interestingly, R1 is duplicated in *G. barbadense* whereas it exists as single copy in *G. hirsutum*, suggesting either a gain in *G. barbadense* or a loss in *G. hirsutum*. Compared to the bordering syntenic block 2 and block 8 (Additional file 7: Figure S3), the small repeats at the ends of R1 that might account for the large duplication event and supply some information on the origin of R1 since the divergence from a common ancestor. It bears noting, however, that the assembled circular map likely represents only one of several possible actual configurations of the genome. Mitochondrial repeats frequently recombine, resulting in an equilibrium composed of multiple configurations (Additional file 7: Figure S3). As both species of cotton probably include several isoforms, differing by repeat-based configurations. The placement of these repeats relative to other syntenic blocks suggest there exists interspecies reorganization during the evolution of *G. barbadense* and *G. hirsutum*. Notably, however, the rearrangements detected between these two mitochondrial genomes did not disrupt gene clusters, which mostly were in syntenic regions. Further sequencing of additional cotton mitochondrial genomes will be necessary to elucidate the extent and fluidity of genomic rearrangements in cotton mitochondrial genomes.

### Nucleotide-level changes in cotton mitochondrial genomes

**Fig. 4:**
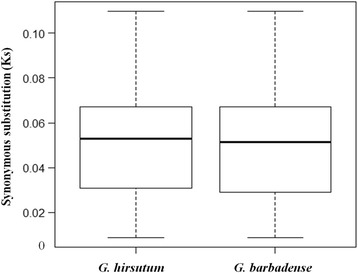
Distribution of Ks values between two cotton mitochondrial genomes and *C. papaya*

Synonymous substitution rates (Ks values) of orthologous gene pairs serve as a useful measure of evolutionary distance [62]. The average Ks values for 35 collinear mitochondrial gene pairs were 0.051 for either *G. barbadense* or *G. hirsutum* versus *C. C. papaya* (Fig. 4), about 1/10^th^ the value for nuclear genes [63]. These data indicate the commonly observed low mutation rates for mitochondrial genes, likely because of efficient DNA repair mechanisms [12, 13]. These data, as well as paired *t*-tests (*P* = 0.957 > 0.05) indicate that the two *Gossypium* mitochondrial genomes have had equal mutation rates. dN/dS ratios for six genes (*nad6*, *ccmB*, *ccmFN*, *sdh3*, *sdh4*, *matR*) in both mitochondrial genomes were greater than 1 (Table 4), suggesting that these genes may have experienced positive selection during divergence from the common ancestor of *Gossypium* and *C. papaya*.

**Table 4:** dS and dN/dS values of 35 genes between two cotton mitochondrial genomes and *C. papaya*

Genes	dS	dS	dN/dS	dN/dS
	*G. hirsutum*	*G. barbadense*	*G. hirsutum*	*G. barbadense*
*nad1*	0.0311	0.0273	0.4950	0.5641
*nad2*	0.0222	0.0222	0.2589	0.2589
*nad3*	0.0671	0.0671	0.5962	0.6574
*nad4*	0.0320	0.0320	0.3857	0.3563
*nad4L*	0.0960	0.0960	0.2296	0.2296
*nad5*	0.0177	0.0177	0.8689	0.8689
*nad6*	0.0087	0.0087	3.1102	3.1102
*nad7*	0.0148	0.0148	0.9031	0.9031
*nad9*	0.0673	0.0673	0.2110	0.2110
*cob*	0.0648	0.0648	0.2121	0.2121
*cox1*	0.0527	0.0527	0.1506	0.1506
*cox2*	0.0528	0.0472	0.2680	0.2993
*cox3*	0.0451	0.0453	0.3832	0.3039
*atp1*	0.0663	0.0663	0.2157	0.2157
*atp4*	0.0555	0.0555	0.8377	0.8377
*atp6*	0.0919	0.0919	0.6743	0.6743
*atp8*	0.0649	0.0649	0.8732	0.8732
*atp9*	0.0570	0.0570	0.4892	0.4892
*ccmB*	0.0162	0.0162	1.4726	1.4726
*ccmC*	0.0659	0.0659	0.4163	0.4163
*ccmFC*	0.0529	0.0501	0.5313	0.6058
*ccmFN*	0.0306	0.0306	1.1463	1.1463
*sdh3*	0.1099	0.1099	1.0637	1.0637
*sdh4*	0.0513	0.0513	1.1205	1.1205
*matR*	0.0176	0.0176	1.5716	1.5716
*mttB*	0.0362	0.0362	0.5901	0.5901
*rpl2*	0.0398	0.0398	0.8391	0.8391
*rpl5*	0.0828	0.0828	0.6046	0.6046
*rpl16*	0.0752	0.0752	0.1628	0.1628
*rps3*	0.0814	0.0814	0.5694	0.5694
*rps4*	0.0718	0.0718	0.7649	0.7649
*rps7*	0.0367	0.0367	0.9328	0.9328
*rps10*	0.0216	0.0216	0.3991	0.3991
*rps12*	0.0851	0.0851	0.2572	0.2572
*rps14*	0.0134	0.0134	0.3387	0.3387

### Pseudogenes in mitochondrial genomes of land plants

**Fig. 5:**
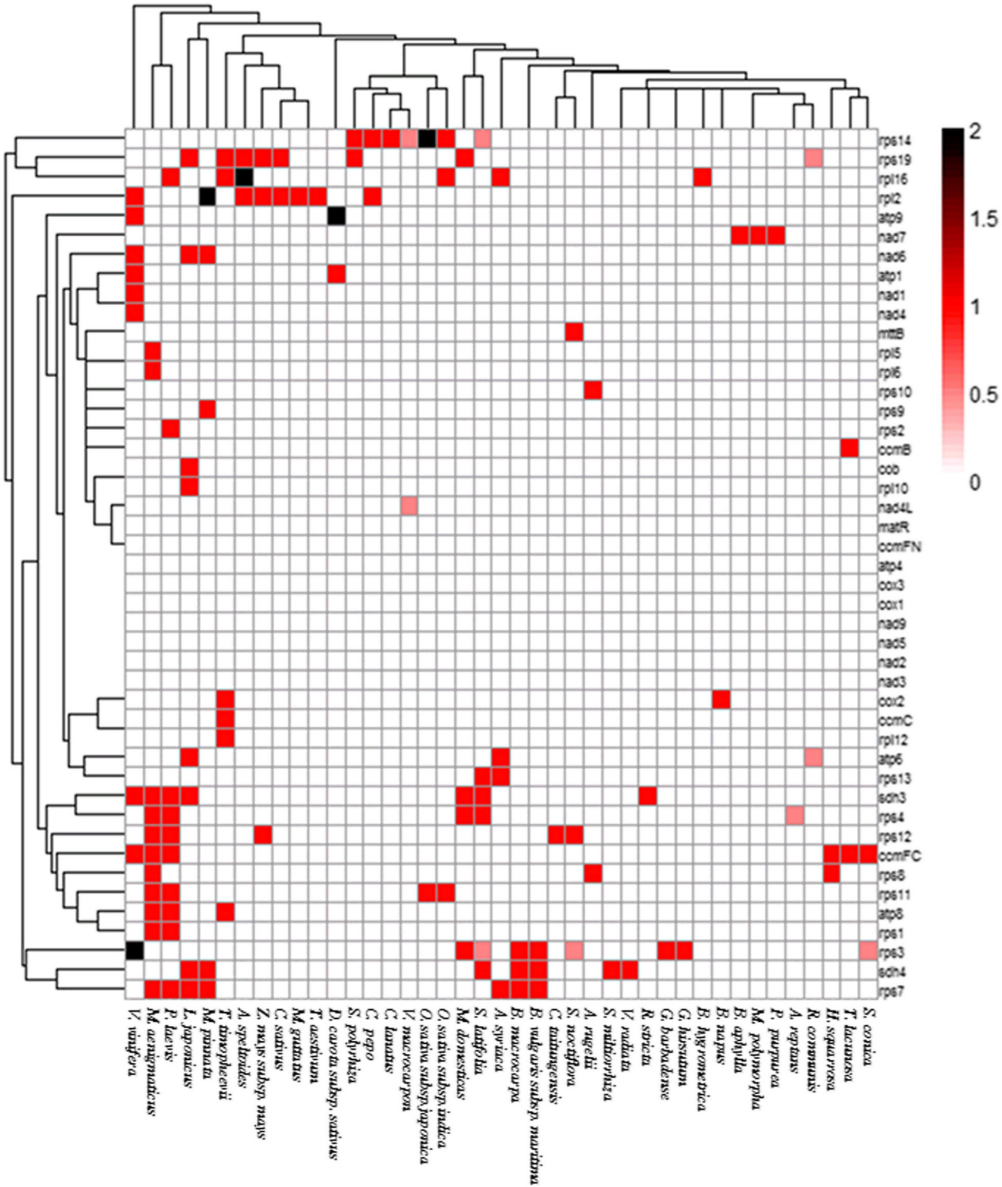
Distribution of pseudogenes in 41 mitochondrial genomes sequenced of land plants. The scale of pseudogene number is depicted on the side, and ranges from 0 copies to 2 copies, with 0.5 representing possible pseudogenes. The numbers 1–41 on the x-axis correspond to the number given to each mitochondrial genome (see Table 5)

As mentioned above, the suite and synteny of genes was largely conserved between *G. barbadense* and *G. hirsutum*. Likewise, both cotton genomes shared the relatively few potential pseudogenes. This is interesting because while complex I, III, IV, and V genes (*nad*, *cox*, *cob*, and *atp* genes, respectively) are generally universally conserved in land plant mitochondrial genomes [37], pseudogenes also are ubiquitous [64, 65]. To explore further the patterns of pseudogenization in mitochondrial genes, we analyzed all 41 currently sequenced mitochondrial genomes deposited in NCBI (Table 5). This comparison revealed that: (1) pseudogenes may arise from any category of mitochondrial genes and from the chloroplast genome; (2) the frequency of pseudogenization (Fig. 5) is highest for ribosomal protein genes, and lower for genes encoding subunits of the respiratory chain proteins. This is consistent with a prior analysis of pseudogene distribution of 41 protein-coding genes among 20 land plants mitochondrial genomes [3], who also reported that pseudogenes mainly occurred in complex II subunit of the respiratory chain (*sdh* genes) and ribosomal protein genes (*rps* genes and *rpl* genes); (3) some pseudogenes are lineage-specific (e.g., in *Oryza sativa subsp. japonica* and *Oryza sativa subsp. indica*, Table 5); and (4) the presence of multi-copy pseudogenes in some mitochondrial genomes (e.g., *rpl16*, *atp9*, *rps3*, etc.), as observed here and in previously [39] (*Vitis vinifera*), may indicate further duplication during pseudogene formation. Recent research has shown that some pseudogenized genes followed endogenous functional gene transfer to the nucleus [37] leading to the gradual mutational degradation of the corresponding mitochondrial copies. In addition, the tendency for ribosomal genes to pseudogenized more frequently may be associated with three sets of translation systems in a single cell compartment that leads to more “gene replacement” [37]. Analysis of additional mitochondrial genomes will help illuminate these trends.

**Table 5:** Pseudogenes in 41 mitochondrial genomes sequenced

No.	Species	Pseudogenes
1	*Marchantia polymorpha*	*nad7*
2	*Pleurozia purpurea*	*nad7*
3	*Treubia lacunosa*	*ccmB*, *ccmFC*
4	*Megaceros aenigmaticus*	*rps1*, *rps4*, *rps7*, *rps8*, *rps11*, *rps12*, *rpl5*, *rpl6*, *atp8*, *ccmFC*, *sdh3*
5	*Phaeoceros laevis*	*rps1*, *rps2*, *rps4*, *rps7*, *rps11*, *rps12*, *rpl16*, *atp8*, *ccmFC*, *sdh3*
6	*Buxbaumia aphylla*	*nad7* ^a^
7	*Anomodon rugelii*	*rps8*, *rps10*
8	*Aegilops speltoides*	*rpl2*, *rpl16*(2), *rps19*
9	*Brassica napus*	*cox2-2*
10	*Ajuga reptans*	*rps4* ^a^
11	*Asclepias syriaca*	*rps7*, *rps13*, *rpl16*, *atp6*
12	*Beta macrocarpa*	*rps3*, *rps7*, *petG*(cp),*sdh4*
13	*Beta vulgaris subsp. maritima*	*rps3*, *rps7*, *petG*(cp),*sdh4*
14	*Boea hygrometrica*	*rpl16*
15	*Citrullus lanatus*	*rps14*
16	*Cucumis sativus*	*rps19*, *rpl2*
17	*Cucurbita pepo*	*rps14*, *rpl2*
18	*Cycas taitungensis*	*rps12*
19	*Daucus carota subsp. sativus*	*atp1*, *atp9*(2)
20	***Gossypium barbadense***	***rps3***
21	***Gossypium hirsutum***	***rps3***
22	*Huperzia squarrosa*	*rps8*, *ccmFC-1*, *ccmFC-2*
23	*Lotus japonicus*	*rps7*, *rps19*, *rpl10*, *cob*, *nad6*, *sdh3*, *sdh4*, *atp6*
24	*Malus x domestica*	*rps3*, *rps4*, *rps19*, *sdh3*
25	*Millettia pinnata*	*rps7*, *rps19*, *rpl2*(2), *sdh4*, *nad6*
26	*Mimulus guttatus*	*rpl2*
27	*Oryza sativa subsp. japonica*	*rps11*, *rps14*(2)
28	*Oryza sativa subsp.indica*	*rps11*, *rps14*, *rpl16*
29	*Rhazya stricta*	*sdh3*
30	*Ricinus communis*	*rps19* ^a^, *atp6* ^a^
31	*Salvia miltiorrhiza*	*ndhB*(cp), *sdh4*
32	*Silene conica*	*rps3* ^a^, *ccmFc*
33	*Silene latifolia*	*rps3* ^a^, *rps4*, *rps13*, *rps14* ^a^, *sdh3*, *sdh4*
34	*Silene noctiflora*	*rps3* ^a^, *rps12*, *mttB-2*
35	*Spirodela polyrhiza*	*rps14*, *rps19*
36	*Triticum aestivum*	*rpl2*
37	*Triticum timopheevii*	*rps19*, *rpl12*, *rpl16*, *atp8*, *cox2-1*, *ccmC*
38	*Vaccinium macrocarpon*	*rps14* ^a^, *nad4L* ^a^
39	*Vigna radiata*	*sdh4*
40	*Vitis vinifera*	*rps3*(2), *rpl2*, *nad1*, *nad4*, *nad6*, *atp1*, *atp9*, *ccmFC*, *sdh3*
41	*Zea mays subsp. mays*	*rps12*,*rps19*, *rpl2*, *rpl23*, *rbcL*(cp), *ndhB*(cp)

^a^possible pseudogenes, *(2)* two copies of pseudogenes, *cp* pseudogenes of chloroplast origin and *Gossypium species* are given in bold

### *rps3* gene transfer in pieces into the *Gossypium* mitochondrial genome

**Fig. 6:**
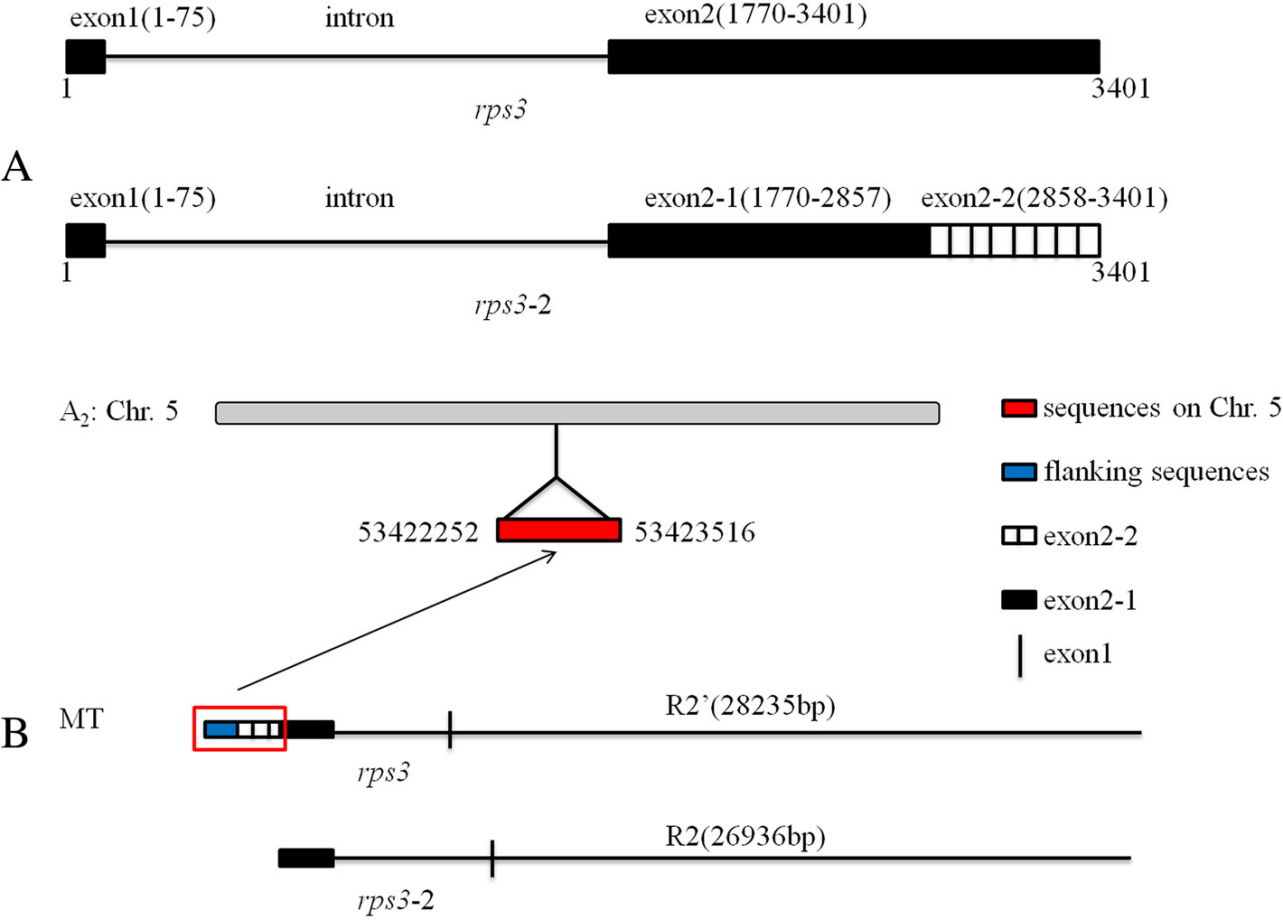
The putative origin mechanism of *rps3*-2 and R2. **a** A structural comparison of *rps3* and *rps3*-2 is shown in the top panel. Exons are depicted as black bars, introns as straight lines, and the striped box indicates the exonic sequence lost in *rps3*-2. Locations for each of the exons are given in parentheses **b** The lower panel illustrates the possible formation mechanism of *rps3*-2 and R2. A_2_: Chr. 5 represents chromosome 5 in the *G. arboreum* genome and MT represents mitochondrial genome of *G. barbadense*, with the top MT graph indicating the arrangement before transferring and the bottom indicating the arrangement after intracellular transferring. The red rectangle and red bar indicate the transferred sequences from the mitochondrial genome to the nuclear genome, respectively. The blue bar represents the flanking sequences transferred along with the latter half of exon 2, depicted again as a striped box. Included in these graphs are the bordering regions between rp3 and R2′ (28,235 bp) and between *rps3*-2 and R2 (26,936 bp)

**Fig. 7:**
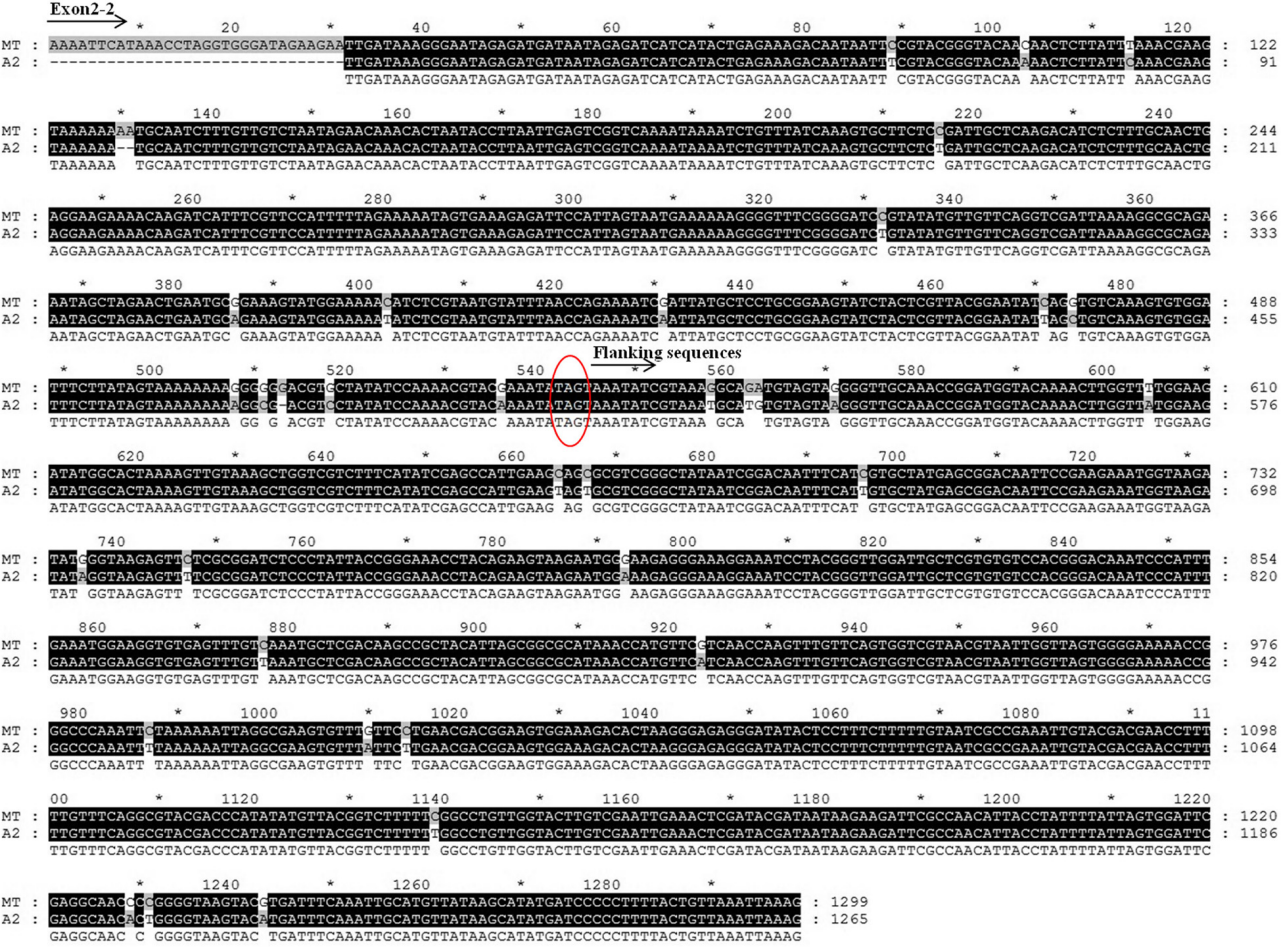
Alignment of rp3 from the *G. barbadense* mitochondrial genome and the corresponding transfer found in the *G. arboreum* nuclear genome. Here, 1–755 bp in the alignment represents the 5′ flanking regions of *rps3*, and 756–1,298 bp consists of exon2-2 from the intact and transferred *rps3* copy, respectively

Like *Vitis, rps3* have partial duplicated copy in the mtDNA of the *Gossypium*. In both the mitochondrial genomes of *G. barbadense* and *G. hirsutum*, there was a duplicated copy of *rps3* (*rps3*-2) which was nearly identical to its corresponding ortholog *rps3.* Horizontal gene transfer (HGT) into mitochondrial genomes is a propensity noted previously [16, 37–39, 66], however, the primary source of the divergent copy of *rps3* is not from HGT but rather from the mtDNA of *Gossypium* itself. The full-length gene of *rps3* in *Gossypium* is 3,401 bp, and contains two exons and one intron (Fig. 6). In both *Gossypium* mitochondrial genomes, however, *rps3*-2 is truncated at the end of the second exon (Fig. 6a). The missing part of this exon in *rps3*-2 was not found elsewhere in either cotton mitochondrial genome, even when using a relaxed BLAST of 1e^−10^ to 1e^−6^. To explore the possibility that the latter half of this exon was copied within the cotton mitochondrial genome and then subsequently migrated to the nucleus, we used the published genomes of *G. raimondii* (D_5_) [67, 68] and *G. arboreum* (A_2_) [69] as BLAST databases (with a cutoff of 1e^−10^). Interestingly, the latter half of exon 2 (exon 2–2; Fig. 6) was recovered from *G. arboreum* chromosome 5 (only), along with 755 bp of additional mitochondrial sequence derived from the flanking region of *rps3* (Figs. 6 and 7). The percent identity between the intact mitochondrial sequence and the nuclear copy is ~97 %, which is similar to the average difference in non-coding regions for nuclear genes in the A- and D- genome cottons. These observations are interesting for two reasons. First, the recovery of this mitochondrial sequence from the *G. arboreum* (A-genome) only, which is also the model maternal progenitor for both *G. barbadense* and *G. hirsutum* [70], suggests that this mitochondria to nuclear transfer occurred subsequent to the divergence of the A- and D- genomes of cotton, which is estimated to have been 5–10 mya; the level of sequence divergence suggests that the transfer occurred shortly after the divergence of the A- and D- lineages. Second, the formation of *rps3*-2 was complex, involving both sequence duplication and intracellular transfer. As shown in Fig. 6, the sequence R2′ (28,235 bp) was duplicated (sequences in red rectangular box in Fig. 6b), including part of *rps3* transferred to nuclear genome, and the remnant sequences of R2′ remained in mitochondrial genome. These remnant sequences became *rps3*-2 and R2, respectively.

### Patterns of tRNA presence in plant mitochondrial genomes

**Fig. 8:**
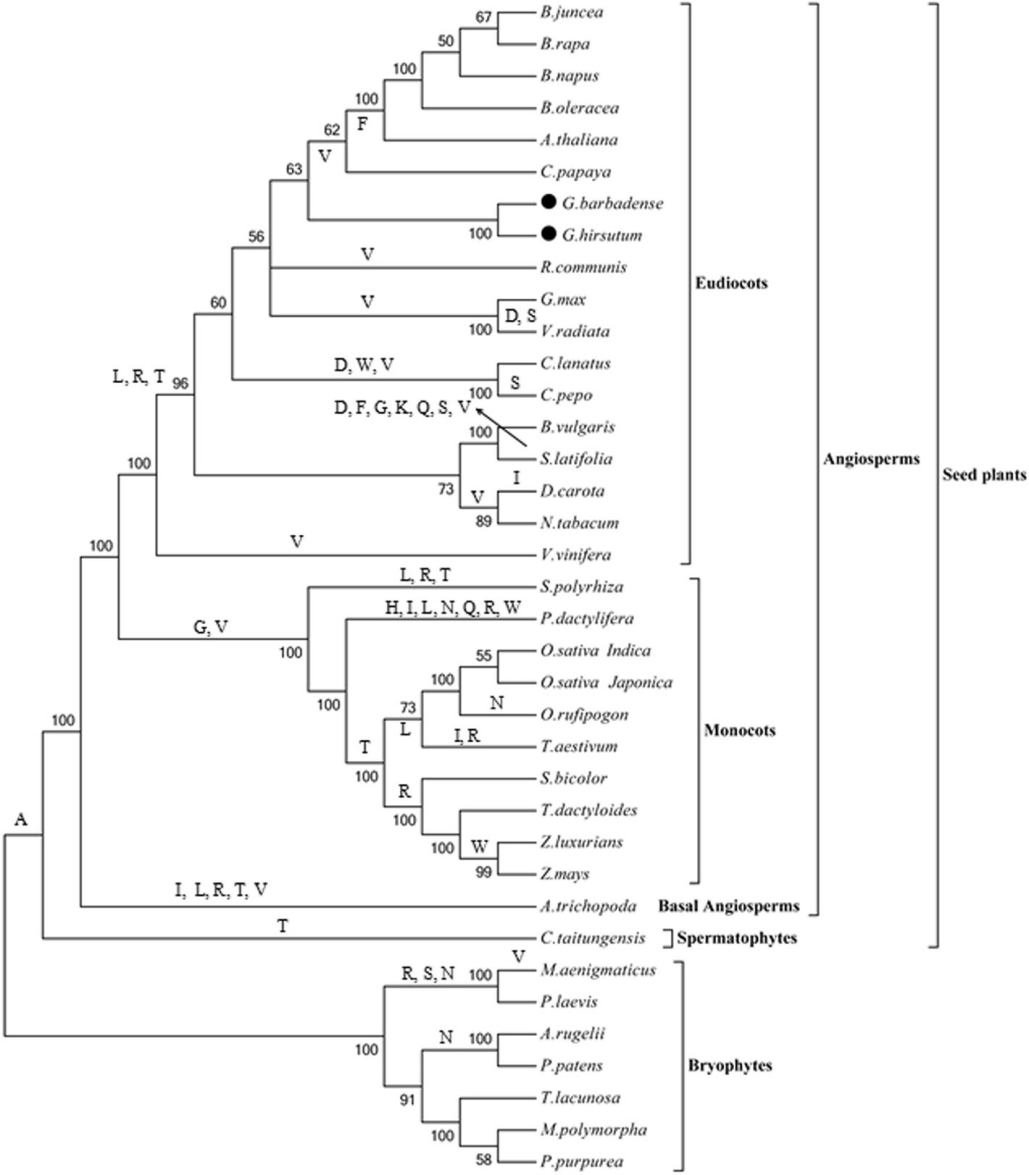
The loss of tRNAs in 37 plant mitochondrial genomes. The phylogenetic tree was constructed based on nucleotide sequences of 17 mitochondrial genes including *nad1*, *nad2*, *nad3*, *nad4*, *nad4L*, *nad5*, *nad6*, *nad9*, *cob*, *cox1*, *cox2*, *cox3*, *atp1*, *atp4*, *atp6*, *atp8* and *atp9* using maximum likelihood (ML) method with the model GTR + G + I in MEGA5.05 [59]. The amino acids at the nodes represent the corresponding tRNA gene losses from mitochondrial genomes, and the arrow stands for tRNA loss in *S. latifolia*

While plant mitochondrial genomes possess native tRNAs, nuclear-encoded tRNAs need to be imported from the cytosol to compensate for those that are missing [71–73]. In both *Gossypium* genomes, four (*trnA*, *trnL*, *trnR* and *trnT*) of the 20 tRNAs are absent from the mitochondrial genome, and therefore must be imported from the cytosol. To evaluate the patterns of loss of tRNAs during the evolution of plant mitochondrial genomes, we analyzed tRNAs in 37 land plant mitochondrial genomes (Fig. 8). Of the genomes analyzed, only the non-seed plants *Marchantia polymorpha*, *Pleurozia purpurea* and *Treubia lacunose* have a complete set of tRNAs. Patterns of presence/absence suggest that *trnA* was lost early in the evolution of seed plants, while t*rnL*, *trnR*, *trnT*, and *trnV* were lost during the evolution of the eudicots. Interestingly, *trnV* exists in both *Gossypium* and *B. vulgaris*; however, these may both represent subsequent gains, as BLAST comparison of the *trnV* copy in *Gossypium* shows more than 99 % identity to the corresponding copy in the *Gossypium* chloroplast (Table 1). Similar to the observation for the eudicots, *trnG* was lost early during monocot evolution. Finally, *S. latifolia* and *P. dactylifera* experienced rapid loss of large numbers of tRNAs [74]. Overall, only *trnC*, *trnE*, *trnM*, *trnP* and *trnY* are present in all species evaluated, indicating that these tRNAs may be most conserved in plant mitochondrial genomes.

## Conclusion

Mitochondrial genomes of plants are evolutionarily intriguing because of their highly conserved genic content and slow rates of genic evolution [11–13], features which contrast sharply with their highly labile genomic structure, genome size, DNA repair mechanisms and recombination induced by different types and origins of repeated sequences. Common evolutionary modifications of mitochondrial genomes include gene loss [75, 76]; intracellular, intergenomic transfers [37, 75, 77, 78]; sequence acquisitions, horizontal transfers from other, sometimes distantly related species [3]; multiple sequence rearrangements [21] and DNA repair mechanisms [11–13]. Here we compare the mitochondrial genomes of two closely related allopolyploid cotton species, which diverged only 1–2 mya and share the same organellar ancestry [70, 79]. Despite the short divergence time separating *G. barbadense* and *G. hirsutum*, many of the hallmark features of mitochondrial genome evolution are evident, including differential genic content, gains/losses of multiple small and large repeats, and genome rearrangements, horizontal transfer, and the evolution of duplicated genes. We illustrate how phylogenetic analysis combined with divergence data can illuminate the timing of duplicated gene formation and of differences in mitochondrial tRNA and protein coding gene content. Increasing insight into the mechanisms and functional consequences of mitochondrial gene and genome variation are expected as additional plant mitochondrial genome sequences become available.

### Availability of supporting data

The data sets supporting the results of this article are included within the article and its additional files.

## Additional files

Additional file 1: Figure S1. The distributions of plastid, mitochondrion and nuclei in sucrose-density gradient. (JPEG 325 kb) Additional file 2: Figure S2. PCR validation for Pima90-53 mtDNA and total DNA with two mitochondrial, nucleus and chloroplast markers, respectively. Note: M1 (*nad4L*) and M2 (*ccmB*) represent mitochondrial markers; N1 (*actin*) and N2 (*RT165*) represent nuclear markers; C1 (*GCS20*) and C2 (*GCS60*) represent chloroplast markers. D: D2000 plus DNA ladder. (JPEG 252 kb) Additional file 3: Table S1. Partial primers of PCR in genome assembling. (DOC 91 kb) Additional file 4: Table S2. Chloroplast-like sequences in the mitochondrial genome of *G. barbadense*. (DOC 37 kb) Additional file 5: Table S3. Repeats (>100 bp) in *Gossypium barbadense* mitochondrial genome. (DOC 59 kb) Additional file 6: Table S4. Eight syntenic blocks (>10 kb) between *Gossypium barbadense* and *G. hirsutum* mitochondrial genomes. (DOCX 14 kb) Additional file 7: Figure S3. Schematic illustration of the eight syntenic regions in mitochondrial genomes of *G. barbadense* and *G. hirsutum* and five repeats located in *G. barbadense* while the sequences of R1 were just present once in the mitochondrial genome of *G. hirsutum*. The map has been rotated 90° counterclockwise after being inverted compared to Fig. 1. (JPEG 285 kb)

## Abbreviations

*Gossypium barbadense*: *G. barbadense*
*Gossypium hirsutum*: *G. hirsutum*
CMS: Cytoplasmic male sterility
ORFs: Open reading frames
rRNAs: Ribosomal RNAs
tRNAs: Transfer RNAs
CTAB: Cetyltrimethyl ammonium bromide
mtDNA: Mitochondrial DNA
LA-PCR: Long and accurate polymerase chain reaction
HGT: Horizontal gene transfer
