# Supplementary figures and images for: Rapid evolutionary divergence of Gossypium barbadense and G. hirsutum mitochondrial genomes
Source: BMC Genomics. 2015 Oct 12;16:770. doi: 10.1186/s12864-015-1988-0 (PMC4603758; doi:10.1186/s12864-015-1988-0)

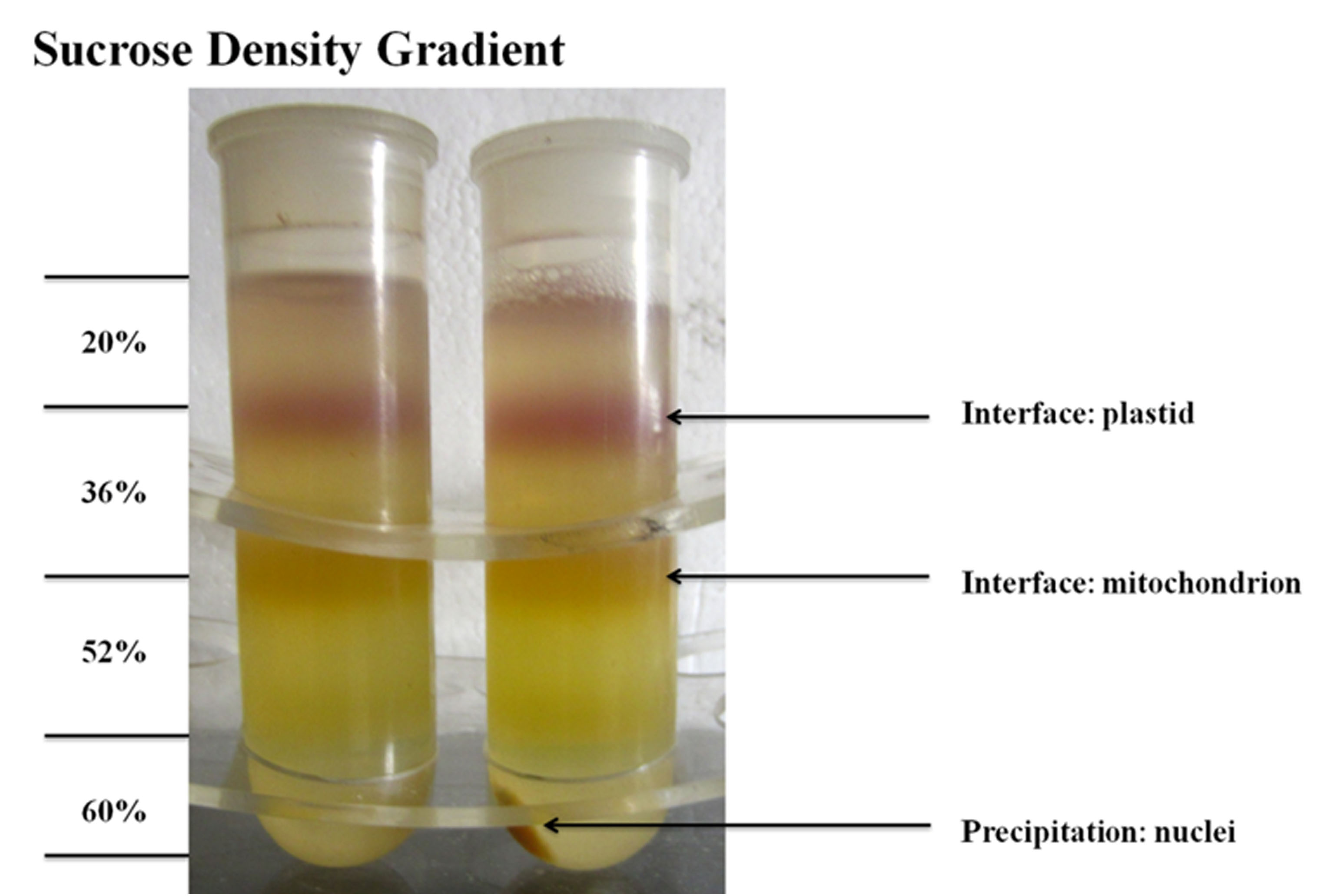

Supplement: Additional file 1: Figure S1. — The distributions of plastid, mitochondrion and nuclei in sucrose-density gradient. (JPEG 325 kb) [file 12864_2015_1988_MOESM1_ESM.jpg]

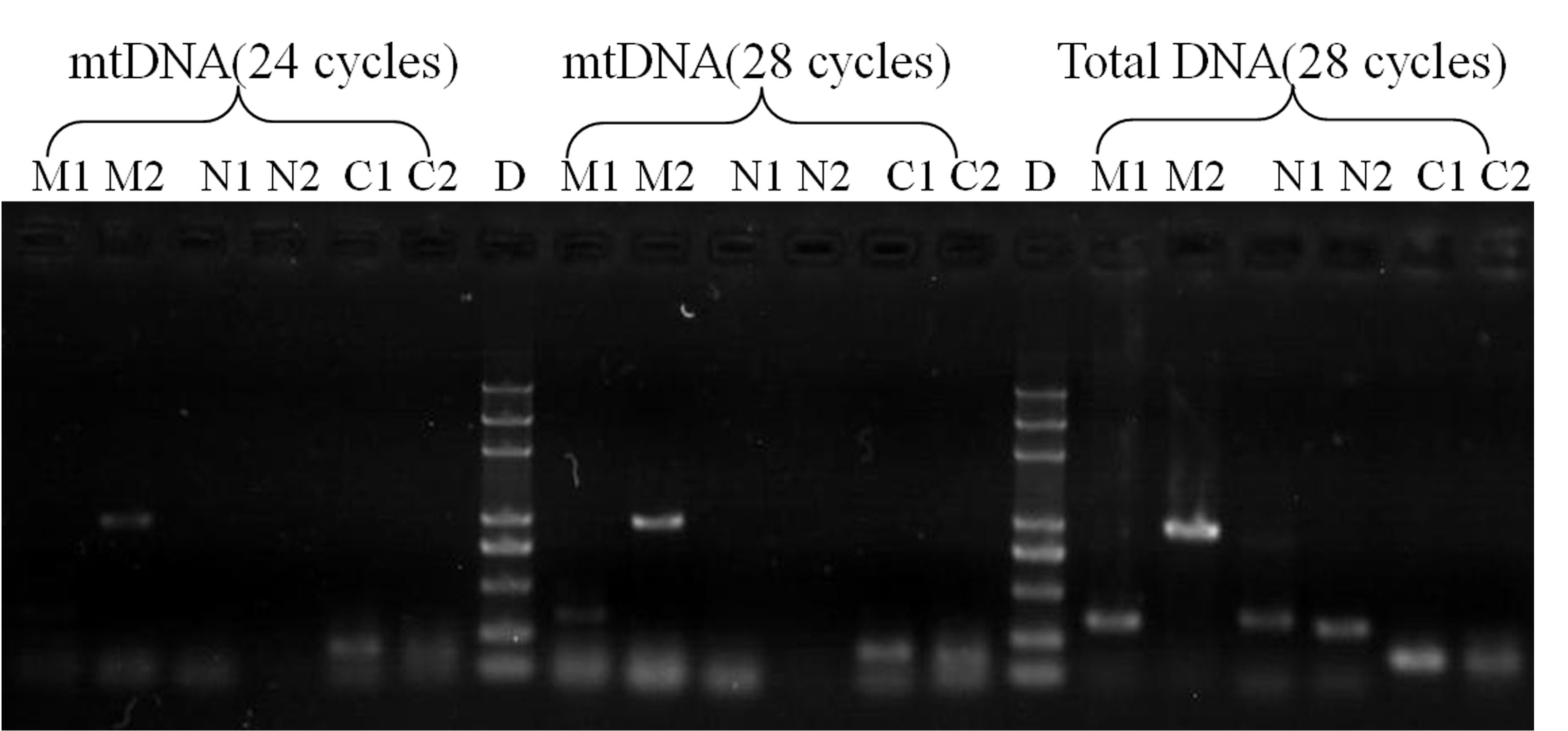

Supplement: Additional file 2: Figure S2. — PCR validation for Pima90-53 mtDNA and total DNA with two mitochondrial, nucleus and chloroplast markers, respectively. Note: M1 (nad4L) and M2 (ccmB) represent mitochondrial markers; N1 (actin) and N2 (RT165) represent nuclear markers; C1 (GCS20) and C2 (GCS60) represent chloroplast markers. D: D2000 plus DNA ladder. (JPEG 252 kb) [file 12864_2015_1988_MOESM2_ESM.jpg]

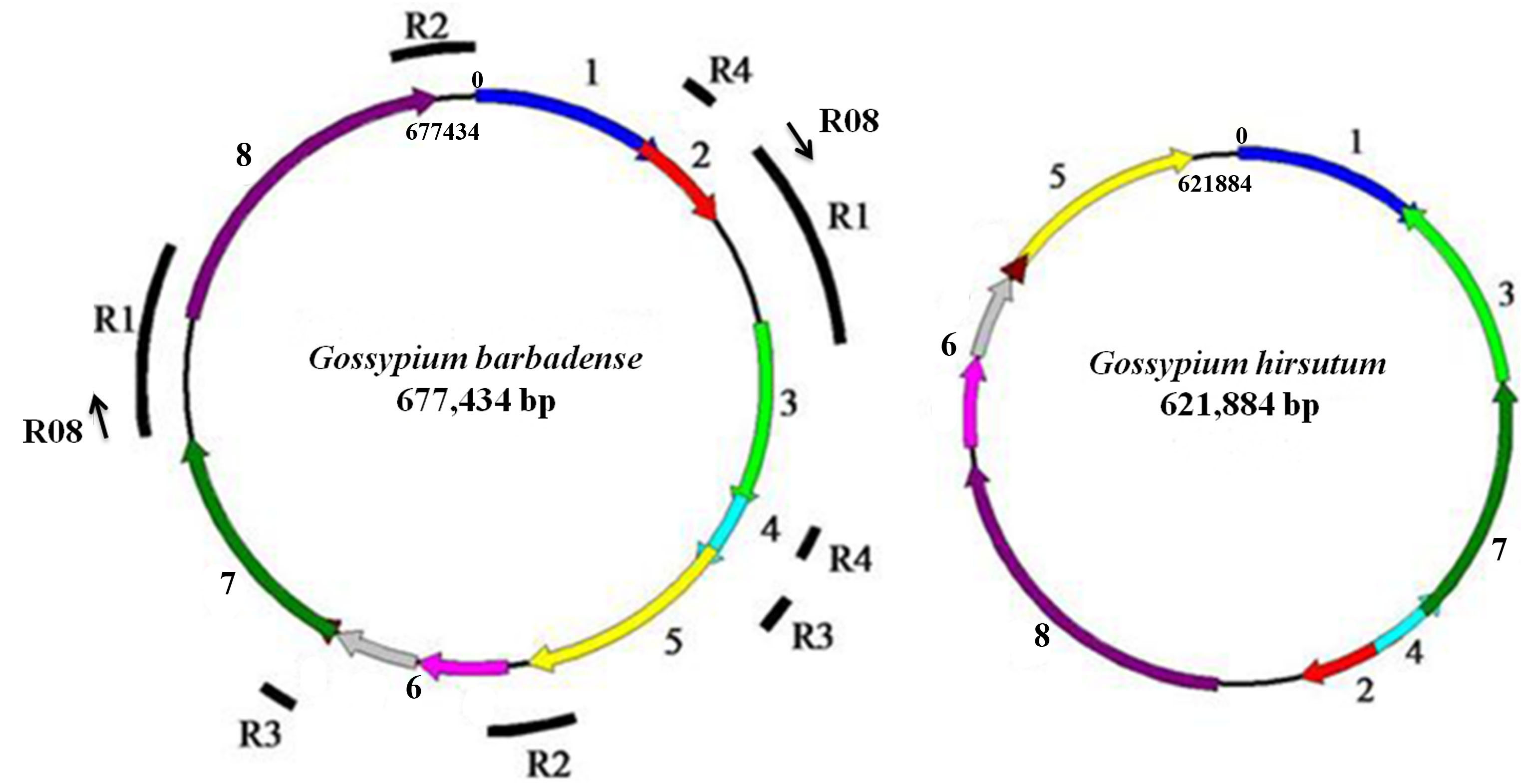

Supplement: Additional file 7: Figure S3. — Schematic illustration of the eight syntenic regions in mitochondrial genomes of G. barbadense and G. hirsutum and five repeats located in G. barbadense while the sequences of R1 were just present once in the mitochondrial genome of G. hirsutum. The map has been rotated 90° counterclockwise after being inverted compared to Fig. 1. (JPEG 285 kb) [file 12864_2015_1988_MOESM7_ESM.jpg]
