# Supplementary material for: Rapid evolutionary divergence of Gossypium barbadense and G. hirsutum mitochondrial genomes
Source: BMC Genomics. 2015 Oct 12;16:770. doi: 10.1186/s12864-015-1988-0 (PMC4603758; doi:10.1186/s12864-015-1988-0)
Supplement: Additional file 3: Table S1. — Partial primers of PCR in genome assembling. (DOC 91 kb) [file 12864_2015_1988_MOESM3_ESM.doc]

**Table S1**

Partial primers of PCR in genome assembling

| Primer name | Forward primer(5'-3') | Reverse primer(5'-3') | Position in genome |
| --- | --- | --- | --- |
| P01 | TTCTCTTCCATACGCCCTAA | CTGTCACCCTCTCATTCTCC | 17,545-17,564; 17,847-17,828 |
| P02 | CGCTCGTGTCCTATTCAGTC | GCATATCGGTTATCGGTTGT | 28,590-28,609; 28,964-28,945 |
| P03 | TAATGGTGAAGACCCAGG | TTGATACGGAACAAAGCC | 31,711-31,728; 32,039-32,022 |
| P04 | TTGGGTCTAATGTTCGGG | AAAAAGGGGTGACGGTGT | 51,122-51,139; 51,463-51,446 |
| P05 | ACATTGACAGAAAGGGGA | AATAATTAAGCAAGCGGC | 53,438-53,455; 53,753-53,736 |
| P06 | AGCAGATAGAGCAAAGGC | AAGAGAAGGGGTGGTAGG | 53,866-53,883; 54,025-54,008 |
| P07 | AAGAAGGAACGGGAAGAC | TCGAACAGAAGAAGAGCT | 62,029-62,046; 62,501-62,484 |
| P08 | AGGATTTCTCGGAGCAAC | TCTCGTCTTCAATGGGGT | 203,581-203,564; 203,564-203,218 |
| P09 | CGCTACGCCTGTCCTAAT | GACGGTGGAACCCCTCTT | 174,685-174,668; 174,337-174,354 |
| P10 | GACTCGTGTCGTGTAGTG | ATCGAAGAAGAGGCAGAT | 159,000-158,983; 158,679-158,696 |
| P11 | ACGAGCAGAAGCGGAGAC | GGATAAACAAGCAAGGGT | 485,180-485,163; 484,897-484,914 |
| P12 | TCTCTTAAGCCCTCCCCA | AACCAATGACTCTTGCCG | 467,224-467,207; 466,817-466,834 |
| P13 | AACGGCCACTGCCTGAAA | GCGACCATGAATCGAACC | 462,775-462,758; 462,334-462,351 |
| P14 | AGGAACTCAAAACCACAA | CTATCACAACAAGGCAAG | 454,876-454,859; 454,482-454,499 |
| P15 | TTCTTTCTCTTTTCGTTT | CTCAATTCTTTGTTTGCT | 449,827-449,810; 449,525-449,542 |
| P16 | TGCTTTGCCTTCCATTTA | TGGCCTGCCTCCTTATAT | 449,388-449,371; 449,100-449,117 |
| P17 | GTTTGAGTGGAAGGTGGG | GGCTTTCTTTCTAATGGG | 448,995-448,978; 448,703-448,720 |
| P18 | GGTCTTCCTCGCTTGCTC | GTCTTCTTCCTGCCTCCC | 444,605-444,588; 444,164-444,181 |
| P19 | TCCTCCCTTCTCTAACAC | TTTCGACCTCACTCACTT | 439,149-439,132; 438,835-438,852 |
| P20 | CAAGCCCACTTTCCCACC | TCCGACATTCACCATCAA | 433,200-433,183; 432,843-432,860 |
| P21 | ACTAAGGAGCGAAAGCGA | GACAAACGACCCAAGACT | 426,624-426,607; 426,126-426,143 |
| P22 | AAAGGTCAACGCAAGGAT | GAAAGGGGGTGAGATAGG | 79,801-79,818; 80,206-80,189 |
| P23 | TTTGGGTAAGAAGAGCGT | TAAAGGGAACTGGAAGGG | 83,651-83,651; 84,089-84,072 |
| P24 | CACTACTGAGACTGGCAC | AATAGGCTCTTAGATGGA | 92,619-92,636; 92,937-92,937 |
| P25 | GTGACACCCCTCCCTCTG | GTTCTTTCGCTTTCCTGG | 100,863-100,880; 101,351-101,334 |
| P26 | CGTAGTTTTCCGCCACCT | CCCTCCCCCTCTCTCTTT | 111,964-111,981; 112,325-112,308 |
| P27 | CGCATTTCATCTTTTTCC | ATTACGCCTTTCCTCCAC | 112,694-112,711; 113,077-113,060 |
| P28 | AAAGGCTGCTGGGATTCA | ATTGCCAAAAGTGGGGGA | 114,007-114,024; 114,559-114,542 |
| P29 | TCGCTTGCTTGCTGTCTA | CCTACTGCATCATTCGCC | 119,161-119,178; 119,178-119,509 |
| P30 | TTCTTGTAGTTCCGCTTC | TGTTTCTCCTTCGTTTGA | 119,509-127,531; 128,093-128,093 |
| P31 | AAAAAAAGAGGTCAGTGC | AATATGATGATGGGAATG | 133,175-133,192; 133,759-133,742 |
| P32 | CTGCCCGCATTTCCTTTT | TGGTTCCTCTGTCGTCGC | 138,815-138,832; 138,980-138,963 |
| P33 | TCGGTTCTTCTTAGTTGG | TATTCTGTGTCCCGTGCT | 139,322-139,339; 139,931-139,931 |
| P34 | CCTGATAGAGGCAGAAGC | TTTGGTCAACGAGACGTA | 140,873-140,890; 141,431-141,414 |
| P35 | ACGAGCGGAGCGAAAACA | CCCGAAACCCCCCAGTAA | 554,144-554,161; 554,592-554,575 |
| P36 | AGTCTGCTCCAACCATCC | TCAATCAGTCGTCCCTCA | 577,485-577,502; 577,722-577,705 |
| P37 | TAAGGTTTGCGATAGATG | AGAAGGAAGGATTGGACT | 581,224-581,241; 581,633-581,616 |
| P38 | TACCACAACTTGGATGAA | CAGACACAGACAGGACAG | 595,428-595,445; 595,812-595,812 |
| P39 | GGACGAAAGAAGGGAAGT | TTTGTTGTGCGAAGAGTG | 615,293-615,310; 615,623-615,606 |
| P40 | CTCTTCAAATCGCCTTAG | GAGTACCCGTATCCTGTC | 626,240-626,257; 626,705-626,688 |
| P41 | TAAGGCTTGTGGCTAAAT | GTACACGAGTACCGATGG | 627,988-628,005; 628,548-628,531 |
| P42 | CTCAAGGCGAATCAAAAC | GAAGAGGAAGGGAAGGAC | 635,663-635,680; 635,971-635,954 |
| P43 | ATCAGATAGTGGGACCTC | GCCTAATAGACTGGAATG | 647,221-647,238; 647,744-647,727 |
| P44 | GTGCTCCATTGTATTCCC | CAGTCCTGCCCACTCTTC | 313,760-313,777; 314,046-314,029 |
| P45 | CCCTTTCCTTTTGTCTGG | GGATTTGATTCCTTTGCC | 314,175-314,192; 314,467-314,450 |
| P46 | TCAATAGGCAATCACACA | GGAAGAGATAGCAGGAAG | 321,798-321,815; 322,153-322,136 |
| P47 | CTCTTCCCTACAACCCAT | TCCATAAACCTAGACCCC | 322,688-322,688; 323,143-323,126 |
| P48 | TGGCAGTCAGTCTGTTCA | AGGGTCTTTTCCTCGATA | 328,102-328,119; 328,375-328,358 |
| P49 | TCGTATCTCCTGCTCGTC | CAGTGGCTCCATTTCTTA | 338,592-338,609; 338,891-338,874 |
| P50 | TCAAGGCGATGAGAGACT | GGACCTGAAGAAGGGAAT | 338,874-350,301; 350,579-350,562 |
| P51 | GCTGAGAAAGGAGAATGC | GGAGGGTGATATGAGGGT | 360,702-360,702; 361,322-361,322 |
| P52 | GGAGGGTGATATGAGGGT | GCGAGTTTCTCACCTTCC | 313,766-313,783; 314,276-314,259 |
| P53 | AAGCCCCTTTCCTTTTGT | GGATTTGATTCCTTTGCC | 314,171-314,171; 314,467-314,450 |
| P54 | CTACCAAAAAAGGATACG | TCAGAAAGAGAGGGACAT | 321,758-321,775; 322,047-322,030 |
| P55 | GTGCTGCTCGCTTTCTCT | ACGACCGGCCTATATTTG | 322,992-323,009; 323,453-323,436 |
| P56 | ATCCGCCCTGCCCTGTAT | TCTCTCGTCGTCCTTCCG | 328,024-328,024; 328,529-328,512 |
| P57 | GCTGCTGCTGGTTATCGT | ATTTCTTACTGCGTCGGG | 338,768-338,785; 338,881-338,864 |
| P58 | TCAAGGCGATGAGAGACT | GGACCTGAAGAAGGGAAT | 350,284-350,301; 350,579-350,562 |
| P59 | GCTGAGAAAGGAGAATGC | GGAGGGTGATATGAGGGT | 360,702-360,719; 361,322-361,305 |
| P60 | TGGTAAAGGGGGCGTGAG | GGTGGGGCTGGTAAGATA | 370,250-370,267; 370,920-370,903 |
| P61 | GATGAACCTGCCTTTTTG | TGAGTCTTTGGAACCTGA | 372,925-372,942; 373,450-373,433 |
| P62 | GTCTTTCCTTATCCGCAG | CTTTATCAACCAACCTCC | 383,589-383,606; 384,152-384,135 |
| P63 | ATGGGGTTTACACCGTCG | TGAATCCTGGCCCTTAGG | 249,989-250,006; 250,499-250,482 |
| P64 | GAAGGAACAAAGAGGACG | GGGGTGTAGCGATAGAAA | 252,412-252,429; 252,429-252,955 |
| P65 | CCCAGAGAGTGCTTTTCC | ACCTTGTTACCATGCCGA | 254,189-254,206; 254,658-254,641 |
| P66 | AGAAGACATACAATCAAA | AATCCTAAAACAAAGAAG | 254,939-254,956; 255,279-255,262 |
| P67 | CTTCCTTTGTCCTTCTCT | CTTGCTGTCTTGACTTCT | 257,525-257,542; 258,052-258,035 |
| P68 | CCGACTACACCTTCTTTT | TACTTGCTTTATTTGCGA | 266,619-266,636; 267,189-267,172 |
| P69 | GAGAAAGCAAAGCAGGAA | GTAAAAGGATTGGGCAAA | 272,872-272,889; 273,437-273,420 |
| P70 | GAAGGGAGGTGGTTATTA | ACACTCTTTTGGGCTAGG | 305,194-305,211; 305,211-305,717 |
| P71 | CATTGTATTCCCTGCCTG | GCGAGTTTCTCACCTTCC | 313,766-313,783; 314,276-314,259 |
| P72 | AGCAACTGAAGACATAAG | AAAGAGTACAGGACCAAG | 314,156-314,156; 314,568-314,551 |
| P73 | GCTACGGCTTTCTGTTGT | GCGTTACCTTGCTCTTGA | 321,583-321,600; 321,992-321,975 |
| P74 | CTCTTCCCTACAACCCAT | TCCATAAACCTAGACCCC | 322,688-322,688; 323,143-323,126 |
| P75 | ATCCGCCCTGCCCTGTAT | TCTCTCGTCGTCCTTCCG | 328,024-328,041; 328,529-328,512 |
| P76 | GTTTACCAAAAACACAGG | AACATACTACAGCCCCAG | 56,232-56,249; 57,001-57,001 |
| P77 | TACCGCCCTCGATCAAAC | GAAAGCGTCCTTCCTTCT | 71,640-71,623; 71,623-71,402 |
| P78 | GAAGTGTTTTCGCTCTCA | TACATTCATGCCTTGTCG | 334,878-334,895; 335,543-335,526 |
| P79 | TACATGGACGGTAGTTGGAG | TGCTAAATGAAATGGAAGGA | 367,309-367,328; 368,058-368,039 |
| P80 | ACCCAGCCGATTCTCAAG | TAGGCACAACGCCATTTA | 397,900-397,917; 399,605-399,588 |
| P81 | GGCTCGCTCCTTACTTTT | ATTCTTTGCCCCTCCTTC | 400,015-400,032; 401,638-401,621 |
| P82 | AAAGGTAAGCAGGGGAGA | GGGGGAAAAAGAAAAGTC | 401,913-401,930; 403,376-403,359 |
| P83 | AACCAGAACGAGGGAAACTT | GATCTCAATCAAAACGGCAA | 490,208-490,227; 491,811-491,792 |
| P84 | TACCTTATCCTTGCCCAT | AGTTCTTACCAACCTCCG | 650,513-650,530; 652,066-652,049 |
| P85 | GGGGAAGTGTTTTCGCTC | TCCGCATACGCATATAGG | 677,382-677,399; 625-608 |
