# Supplementary material for: Rapid evolutionary divergence of Gossypium barbadense and G. hirsutum mitochondrial genomes
Source: BMC Genomics. 2015 Oct 12;16:770. doi: 10.1186/s12864-015-1988-0 (PMC4603758; doi:10.1186/s12864-015-1988-0)
Supplement: Additional file 4: Table S2. — Chloroplast-like sequences in the mitochondrial genome of G. barbadense. (DOC 37 kb) [file 12864_2015_1988_MOESM4_ESM.doc]

**Table S2**

**Chloroplast-like sequences in the mitochondrial genome of *G. barbadense***

| NO. | Length (bp) | Identity (%) | Location in mitogenome | Composition |
| --- | --- | --- | --- | --- |
| 1 | 2,203 | 99 | 366,886..369,072 | non-coding sequences |
| 2 | 1,152 | 99 | 56,163..57,314 | non-coding sequences |
| 3 | 266 | 94 | 433,341..433,604 | non-coding sequences |
| 4 | 223 | 96 | 385,394.. 385,616 | non-coding sequences |
| 5 | 201 | 99 | 596,791 ..596,991 | non-coding sequences |
| 6 | 146 | 92 | 433,211..433,354 | trnV-GAC |
| 7 | 152 | 89 | 418,021..418,171 | non-coding sequences |
| 8 | 199 | 84 | 359,289.. 359,487 | trnS-GGA |
| 9 | 110 | 93 | 372,054..372,162 | trnW-CCA |
| 10 | 84 | 99 | 442,479 ..442,562 | trnW-CCA |
| 11 | 84 | 98 | 539,631..539,714 | trnD(GTC) |
| 12 | 84 | 98 | 143,420..143,503 | trnD(GTC) |
| 13 | 80 | 98 | 6,345.. 6,424 | trnH(GTG) |
| 14 | 84 | 95 | 37,464..37,547 | trnN(GTT) |
| 15 | 79 | 94 | 564,649..564,727 | trnM(CAT) |
| 16 | 78 | 94 | 466,795..466,872 | trnM(CAT) |
| 17 | 36 | 100 | 82,369..82,404 | non-coding sequences |
| 18 | 87 | 82 | 554,997..555,083 | ccmC |
| 19 | 35 | 97 | 193,773..193,807 | non-coding sequences |
