# Supplementary material for: Rapid evolutionary divergence of Gossypium barbadense and G. hirsutum mitochondrial genomes
Source: BMC Genomics. 2015 Oct 12;16:770. doi: 10.1186/s12864-015-1988-0 (PMC4603758; doi:10.1186/s12864-015-1988-0)
Supplement: Additional file 5: Table S3. — Repeats (>100 bp) in Gossypium barbadense mitochondrial genome. (DOC 59 kb) [file 12864_2015_1988_MOESM5_ESM.doc]

**Table S3**

**Repeats ( >100 bp) in *Gossypium barbadense* mitochondrial genome**

| No. | Size(bp) | Identity(%) | Copy1 | Copy2 | Copy3 | Copy4 | Type |
| --- | --- | --- | --- | --- | --- | --- | --- |
| R01 | 63,904 | 100 | 94,020-157,923 | 490,232-554,135 |  |  | DR |
| R02 | 26,936 | 100 | 307,992-334,927 | 650,499-677,434 |  |  | DR |
| R03 | 10,615 | 100 | 235,738-246,352 | 393,259-403,873 |  |  | DR |
| R04 | 10,246 | 100 | 64,934-75,179 | 223,609-213,364 |  |  | IR |
| R05 | 495 | 99 | 1-495 | 334,928-335,422 |  |  | DR |
| R06 | 395 | 98 | 245,199-245,593 | 402,720-403,114 | 634,289-634,683 |  | DR/DR |
| R07 | 349 | 99 | 206,756-207,104 | 245,183-244,835 | 402,704-402,356 |  | IR/IR |
| R08 | 293 | 94 | 96,120-96,412 | 326,288-326,580 | 492,332-492,624 | 668,795-669,087 | DR/DR/DR |
| R09 | 257 | 100 | 90,972-91,228 | 244,526-244,782 | 402,047-402,303 |  | DR/DR |
| R10 | 229 | 100 | 40,000-40,228 | 168,219-168,447 |  |  | DR |
| R11 | 223 | 84 | 95,610-95,832 | 377,870-377,648 | 491,822-492,044 |  | IR/DR |
| R12 | 208 | 99 | 77,699-77,906 | 644,412-644,205 |  |  | IR |
| R13 | 194 | 100 | 71,429-71,622 | 217,114-216,921 | 640,755-640,948 |  | IR/DR |
| R14 | 175 | 100 | 366,630-366,804 | 481,243-481,069 |  |  | IR |
| R15 | 173 | 95 | 245,772-245,944 | 310,792-310,964 | 403,293-403,465 | 653,299-653,471 | DR/DR/DR |
| R16 | 167 | 98 | 357,338-357,504 | 641,137-640,971 |  |  | IR |
| R17 | 158 | 95 | 127,493-127,650 | 466,480-466,637 | 523,705-523,862 |  | DR/DR |
| R18 | 143 | 93 | 466,771-466,913 | 564,626-564,768 |  |  | DR |
| R19 | 143 | 94 | 249,626-249,768 | 592,596-592,454 |  |  | IR |
| R20 | 138 | 90 | 206,206-206,343 | 309,730-309,867 | 652,237-652,374 |  | DR/DR |
| R21 | 138 | 96 | 102,303-102,440 | 247,647-247,510 | 498,515-498,652 |  | IR/DR |
| R22 | 129 | 98 | 77,748-77,876 | 366,548-366,676 | 644,363-644,235 |  | DR/IR |
| R23 | 123 | 97 | 124,893-125,015 | 201,696-201,818 | 521,105-521,227 |  | DR/DR |
| R24 | 119 | 97 | 71,355-71,473 | 217,188-217,070 | 373,794-373,676 |  | IR/IR |
| R25 | 118 | 99 | 126,857-126,974 | 443,458-443,575 | 523,069-523,186 |  | DR/DR |
| R26 | 113 | 94 | 268,741-268,853 | 638,753-638,641 |  |  | IR |
| R27 | 106 | 92 | 180,304-180,409 | 238,748-238,643 | 396,269-396,164 |  | IR/IR |
