# Supplementary material for: Rapid evolutionary divergence of Gossypium barbadense and G. hirsutum mitochondrial genomes
Source: BMC Genomics. 2015 Oct 12;16:770. doi: 10.1186/s12864-015-1988-0 (PMC4603758; doi:10.1186/s12864-015-1988-0)
Supplement: Additional file 6: Table S4. — Eight syntenic blocks (>10 kb) between Gossypium barbadense and G. hirsutum mitochondrial genomes. (DOCX 14 kb) [file 12864_2015_1988_MOESM6_ESM.docx]

**Table S4**

**Eight syntenic blocks ( >10 kb) between** ***Gossypium barbadense* and** ***G. hirsutum* mitochondrial genomes**

| Blocks | AD_2_-position | AD_1_-position | Length in AD_2_ (bp) | Length in AD_1_ (bp) | Identity (%) |
| --- | --- | --- | --- | --- | --- |
| 1 | 427-75,187 | 1-74,754 | 74,761 | 74,754 | 99.95 |
| 2 | 64,934-105,391 | 247,693-288,152 | 40,458 | 40,460 | 99.93 |
| 3 | 146,743-223,608 | 141,375-64,505 | 76,866 | 76,871 | 99.91 |
| 4 | 213,356-246,351 | 257,949-224,949 | 32,996 | 33,001 | 99.88 |
| 5 | 235,868-319,573 | 522,321-605,977 | 83,706 | 83,657 | 99.85 |
| 6 | 326,661-403,752 | 455,804-532,682 | 77,092 | 76,878 | 99.89 |
| 7 | 393,258-487,463 | 235,564-141,367 | 94,206 | 94,198 | 99.93 |
| 8 | 531,949-663,501 | 318,489-450,003 | 131,553 | 131,515 | 99.90 |
